# Breed Differences in PCV2 Uptake and Disintegration in Porcine Monocytes

**DOI:** 10.3390/v10100562

**Published:** 2018-10-15

**Authors:** Ruifang Wei, Ivan Trus, Bo Yang, Liping Huang, Hans J. Nauwynck

**Affiliations:** 1 Laboratory of Virology, Faculty of Veterinary Medicine, Ghent University, Salisburylaan 133, B-9820 Merelbeke, Belgium; ruifang.wei@ugent.be (R.W.); ivan.trus@gmail.com (I.T.); bo.yang@ugent.be (B.Y.); 2 Division of Swine Infectious Diseases, State Key Laboratory of Veterinary Biotechnology, Harbin Veterinary Research Institute, The Chinese Academy of Agricultural Sciences, Maduan Street 427, Harbin 150001, China; huangliping@caas.cn

**Keywords:** porcine circovirus type 2, blood monocytes, uptake, disintegration, viral capsids, viral genomes, breeds

## Abstract

Porcine circovirus type 2 (PCV2) is associated with various diseases which are designated as PCV2-associated diseases (PCVADs). Their severity varies among breeds. In the diseased pigs, virus is present in monocytes, without replication or full degradation. PCV2 entry and viral outcome in primary porcine monocytes and the role of monocytes in PCV2 genetic susceptibility have not been studied. Here, virus uptake and trafficking were analyzed and compared among purebreds Piétrain, Landrace and Large White and hybrid Piétrain × Topigs20. Viral capsids were rapidly internalized into monocytes, followed by a slow disintegration to a residual level. PCV2 uptake was decreased by chlorpromazine, cytochalasin D and dynasore. The internalized capsids followed the endosomal trafficking pathway, ending up in lysosomes. PCV2 genome was nicked by lysosomal DNase II in vitro, but persisted in monocytes in vivo. Monocytes from purebred Piétrain and the hybrid showed a higher level of PCV2 uptake and disintegration, compared to those from Landrace and Large White. In conclusion, PCV2 entry occurs via clathrin-mediated endocytosis. After entry, viral capsids are partially disintegrated, while viral genomes largely escape from the pathway to avoid degradation. The degree of PCV2 uptake and disintegration differ among pig breeds.

## 1. Introduction

As a member of the family *Circoviridae*, porcine circovirus type 2 (PCV2) has a single-stranded, circular DNA genome, encoding two major open reading frames (ORFs) [1,2,3]. ORF1 encodes the replicase proteins (Rep and Rep’), crucially initiating the viral DNA replication [4]; ORF2 encodes the immunogenic capsid protein (Cap), solely structuring the non-enveloped, icosahedral viral shell outside [5]. Although being small-sized and simply-structured, PCV2 is associated with various syndromes, including postweaning multisystemic wasting syndrome (PMWS), respiratory disease complex, reproductive disorders, and enteric diseases. These syndromes are collectively designated PCV2-associated diseases (PCVADs), which have a severe impact on the world swine industry [6,7].

PCV2 infection is characterized by replication in lymphocytes, lymphocyte depletion and monocyte infiltration in lymphoid tissues [8,9]. In the cytoplasm of infiltrating monocytes, large amounts of PCV2 antigens and nucleic acids can be demonstrated [9,10,11,12,13,14,15]. This suggests an effective uptake of PCV2 by cells of the monocytic lineage. However, monocytic cells do not support a fully productive infection [11,16]. After migrating into the lymphocyte-depleted follicles, they mainly take up and destroy virus particles, however they do so without a complete disintegration of the virions [9,17,18]. Therefore, their ability to take up and clear the virus from the body may be a critical step in protecting the animal from disease. Although there are some studies on the internalization and disassembly of PCV2 in the artificial monocytic cell line 3D4/31 or in the porcine blood mononuclear cells (PBMC) [11,16,19], PCV2 entry and the viral outcome in primary porcine monocytes have not been examined so far. Moreover, it is reported that the severity of PCVADs differs among breeds, indicating the role of the genetic background of the pigs in PCV2 susceptibility [20,21,22]. Nevertheless, the mechanism involved is poorly understood.

In the present study, it was hypothesized that the level of PCV2 uptake and disintegration in monocytes may differ between breeds and contribute to a genetic difference in clinical outcome. To this end, the fate of PCV2 (viral capsids and genome) in blood monocytes was determined by doing time-course experiments and analysis with confocal microscopy and qPCR. With specific chemical inhibitors, the endocytic pathway was identified. After the uptake, colocalization studies were performed to uncover the trafficking pathway of PCV2. Finally, the capacity of monocytes to take up and lyse PCV2 particles was compared among different pig breeds, in order to find out if genetic differences exist.

## 2. Materials and Methods

### 2.1. Preparation of Peripheral Blood Monocytes

**Figure 1:**
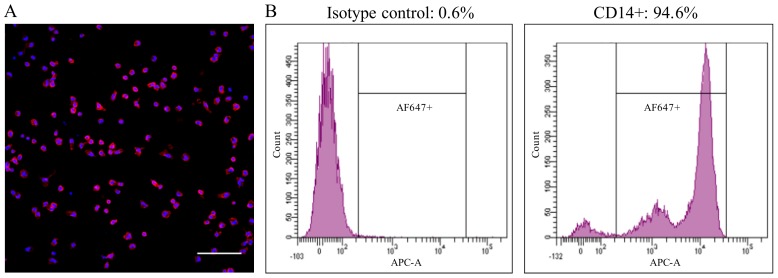
The adherent cell population consisted of >90% of CD14+ cells, as assessed by immunofluorescence staining (**A**) and flow cytometry (**B**). Bar = 100 µm.

Healthy pigs between 6 and 12 weeks-old were used as blood donors. The collection of blood was approved by the ethical committee of Ghent University (EC2013/97). Blood was collected by jugular venipuncture in heparinized tubes and diluted in an equal volume of Dulbecco’s PBS (DPBS) without calcium or magnesium (Gibco, Paisley, UK). PBMCs were isolated by density gradient centrifugation on Ficoll-Paque (1.077 g/mL) (GE Healthcare, Life Sciences, Chicago, IL, USA) at 2100 rpm for 45 min at 18 °C. The interphase cells, containing the PBMCs, were collected and washed three times with DPBS. The cells were resuspended in leukocyte medium based on RPMI-1640 (Gibco), supplemented with 5% fetal calf serum (FCS, Gibco), 100 U/mL penicillin (Continental Pharma, Puurs, Belgium), 0.1 mg/mL streptomycin (Certa, Braine 1’Alleud, Belgium) and 1 µg/mL gentamycin (Gibco). Afterwards, the cells were seeded with a volume of 1 mL per well on 24-well plates (Nunc A/S, Rochester, NY, USA) at a concentration of 2 × 10^6^ cells/mL and cultured at 37 °C with 5% CO_2_. After 18 h, the non-adhering cells were removed by washing three times with RPMI-1640 media. The adherent cell population consisted of >90% of CD14+ cells, as assessed by fluorescence microscopy and flow cytometry (Figure 1). The immunofluorescence staining was performed using a mouse-anti-porcine CD14 mAb (Clone MIL2, 1:100, IgG2b) against monocytes/macrophages, followed by Alexa Fluor 594-conjugated (for fluorescence microscopy) or Alexa Fluor 647-conjugated (for flow cytometry) goat-anti-mouse IgG2b antibodies (1:200; Life Technologies, Carlsbad, CA, USA).

### 2.2. Virus Preparation and Particle Quantification

**Figure 2:**
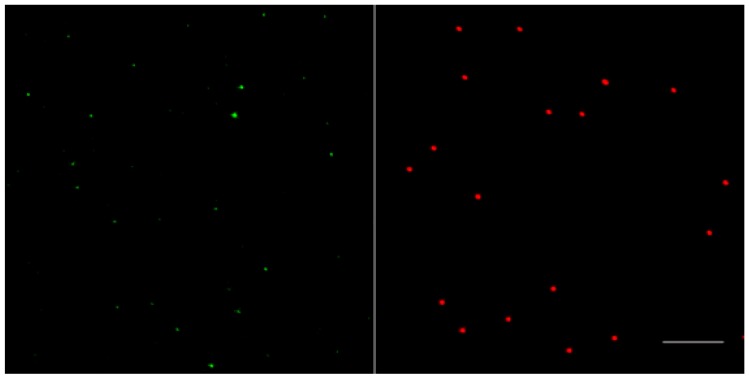
Representative fluorescent confocal images of immunostained PCV2 particles in green and 200 nm red-fluorescent carboxylate-modified microspheres acquired at the same magnification. Bar = 5 µm.

PCV2a strain 1121 (GeneBank accession #AJ293868) isolated from aborted fetuses in Canada was used in this study. For all infection experiments, a virus stock propagated in PK-15 cells was filtered through a 0.45 µm filter to remove cell components and large aggregates of virus particles. To quantify virus particles in the stock, an equal volume of filtered PCV2a-1121 and 200 nm red-fluorescent carboxylate-modified microspheres (10^9^ particles/mL) were mixed. Afterwards, the mixtures were smeared onto microscopic slides, air-dried, and fixed with 4% paraformaldehyde (PF) in PBS at room temperature (RT) for 10 min. After fixation, PCV2 particles were stained with a mouse mAb against the capsid protein (94H8, IgG2a) [23], followed by Alexa Fluor 488-conjugated goat-anti-mouse IgG2a antibodies (1:200, Life Technologies) for 1 h at 37 °C. Afterwards, stained PCV2 particles and fluorophores were mounted with a glycerol solution containing 1,4-diazabicyclo (2.2.2) octane (DABCO) anti-fading agent. Digital images of stained PCV2 particles and fluorophores were acquired at the same magnification, using a Leica TCS SPE laser-scanning spectral confocal microscope, and their distribution is shown in Figure 2. The number of stained PCV2 particles and fluorophores were counted from 20 randomly selected fields. Finally, the number of virus particles in the virus stock was calculated in proportion to the number of the fluorophores.

### 2.3. The Fate of PCV2 Capsids in Blood Monocytes

To understand what happens with PCV2 virions in monocytes, a time-course experiment was performed. Briefly, 1.5 × 10^9^ PCV2 particles were added to 2 × 10^5^ monocytes in each well of a 24-well plate. For short incubation periods (0 min, 15 min, 30 min, 60 min), virus was added and was washed away at each time point, followed by a fixation and a permeabilization of the cells. For periods longer than 1 h (2 h, 3 h, 6 h, 12 h, 24 h, 48 h, 72 h), virus was added and then was washed away after 1 h. Afterwards, cells were supplemented with fresh media, and were further cultured until the indicated time points when they were fixed and permeabilized.

To quantify the amount of capsid proteins inside the cells at each time point, a double immunofluorescence staining was performed. PCV2 antigens were stained as described in Section 2.2, while monocytes were stained with mAbs against CD14 (1:100, IgG2b), in combination with an Alexa Fluor 594-conjugated goat-anti-mouse IgG2b antibody (1:200). The cell nuclei were counterstained with Hoechst 33342 for 10 min at RT. Cells were then mounted and analyzed under the confocal microscope. For each sample, 10 random images were taken. The percentage of PCV2 capsid positive cells was calculated from these images, based on the number of cells with viral particles inside the cells and the total number of cells. The amount of PCV2 capsids within individual CD14+PCV2+ cells from these 10 fields was quantified with ImageJ by measuring the fluorescing area of the green signals inside cells.

### 2.4. Effect of Different Entry Inhibitors on PCV2 Uptake

Blood monocytes were pre-treated for 30 min at 37 °C with different active concentrations of the following endocytic inhibitors: 100 µM amiloride dissolved in DMSO, 10 mM methyl-β-cyclodextrin (mβCD) dissolved in media, 5 µg filipin III mL^−1^ dissolved in DMSO, and 1 µM chlorpromazine dissolved in PBS, 10 µM cytochalasin D dissolved in DMSO, 100 µM dynasore dissolved in DMSO. All chemicals were purchased from Sigma-Aldrich (St. Louis, MO, USA). 1.5 × 10^9^ PCV2 particles were then added to the cells in the presence of the same inhibitors for 1 h to allow the uptake of virus particles. Afterwards, cells were washed to remove the virus-inhibitor mixture, followed by a fixation of cells with 4% PF, and a permeabilization with 0.1% TritonX-100 at RT for 10 min. The active concentration of inhibitors was tested to ensure that the viability of cells was always >90%, as assessed by ethidium monoazide (EMA) staining.

To quantify the level of the PCV2 uptake by monocytes, PCV2 capsids and cells were visualized by a double immunofluorescence staining, followed by analyses using confocal microscopy and ImageJ, as described earlier. For quantification reasons, PCV2 particles that were inside the cells were scored as taken-up by the cell. The average PCV2-uptake level in the different inhibitor groups was expressed as the relative percentage to that of the control group (without inhibitor).

### 2.5. PCV2 Trafficking in Blood Monocytes

A time-course experiment was performed as outlined in Section 2.3. At each time point, cells were fixed with 4% PF and permeabilized with 0.1% TritonX-100. Then, double stainings against different cell markers and the viral capsid protein were performed. To visualize early-endosomes and late-endosomes, cells were stained with goat polyclonal anti-early endosome antigen (EEA-1) antibodies (1:100, Santa Cruz Biotechnology, Dallas, TX, USA) or with goat polyclonal anti-Rab7 antibodies (1:100, Santa Cruz Biotechnology), in combination with Alexa Fluor 594-conjugated rabbit-anti-goat IgG antibodies (1:200, Life Technologies). To visualize lysosomes, cells were stained with rabbit polyclonal anti-LAMP-1 antibodies (1:100, Santa Cruz Biotechnology), followed by Texas Red-conjugated goat-anti-rabbit IgG antibodies (1:100, Life Technologies). To visualize virus particles, PCV2 capsid protein was stained as described earlier in Section 2.2. The cell nuclei were counterstained, after which samples were mounted and analyzed under the confocal microscope. The percentage of PCV2 positive cells showing colocalization with each cell marker was calculated from 10 randomly selected regions at each time point.

### 2.6. The Fate of PCV2 Viral Genomes in Blood Monocytes

A time-course experiment was performed as outlined in Section 2.3. At each time point, cells were scraped and pelleted at 600× *g* for 10 min. Afterwards, the cellular DNA was extracted using QIAamp Cador Pathogen mini kit (Qiagen, Hilden, Germany) following the manufacturer’s instructions. To quantify the copies of viral genomes inside cells, a qPCR with a product of 226 bp in the ORF1 gene region was performed. Briefly, amplifications were conducted in duplicate using specific primers (fwd: 5′-TGG GGC CAC CTG GGT GTG-3′; rev: 5′-GCC AAA AAA GGT ACA GTT CCA CC-3′) and a specific probe (5′-/6-FAM/AGC AAA TGG/ZEN/GCT GCT AAT TTT GCA GAC/IBFQ/-3′) (IDT). The qPCR was performed with an Applied Biosystems StepOnePlus Real-Time PCR System (Applied Biosystems Inc., Foster, CA, USA) using PrecisionPLUS MasterMix (Primerdesign, Southampton, UK).

To further investigate whether the PCV2 genome is present in monocytes as full length or digested fragments, a conventional PCR was performed with the primer set wgPCV2-fw/rev (Table 1) which can amplify the full length genome. The DNA extracted at different time points was used as a template. In detail, the PCR amplification was carried out in a 25 µL reaction, containing 5 µL of 5× PCR Buffer, 0.25 µL of 100 mM dNTPs, 1 µL of 10 µM primers each, 2 µL of DNA template, 0.5 µL of Herculase II fusion DNA polymerase, and 15.25 µL water. The thermal cycling program for the PCR reaction was 94 °C for 2 min, then 30 cycles of 94 °C for 15 s, 51 °C for 30 s, and 68 °C for 2 min, followed by 68 °C for 10 min. The amplification products were examined by agarose gel electrophoresis.

### 2.7. In Vitro Test of the Ability of Lysosomal Deoxyribonuclease II (DNase II) to Degrade PCV2 Genome

**Figure 3:**
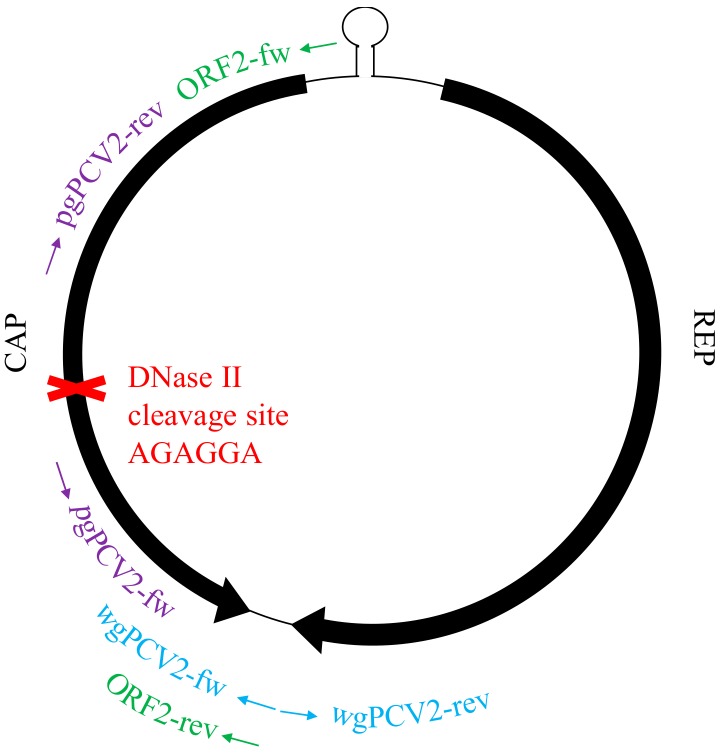
Localization of three primer sets on the circular PCV2 genome with the putative cleavage site of DNase II.

Viral DNA was extracted from a stock of PCV2 strain 1121, using QIAamp Cador Pathogen mini kit (Qiagen). Then, 1 µg of virus DNA was incubated with 100 units of the lysosomal DNase II (Sigma-Aldrich), in the presence of 50 mM sodium-acetate (pH 5.0) at 37 °C for 18 h to allow a complete reaction [24]. In addition, the Mung Bean Nuclease (New England BioLabs, Ipswich, MA, USA) which targets single-stranded DNA, and the Benzonase endonuclease (Millipore, Burlington, MA, USA) which degrades all forms of DNA and RNA, were used to control the degradation of PCV2 genome. Due to the small amount of DNA, instead of a direct visualization by agarose gel electrophoresis, the digested products were purified and evaluated by PCR with three sets of primers (Table 1). These primers amplified different regions of the PCV2 genome, as shown in Figure 3 [23,25]. The PCR was performed with Herculase II fusion DNA polymerase as described in Section 2.6. The amplification products were examined by agarose gel electrophoresis.

### 2.8. Kinetics of PCV2 Uptake and Disintegration in Blood Monocytes from Four Pig Breeds

A total of 14 pigs from four pig breeds (three from the hybrid Piétrain × Topigs20, five from purebred Piétrain, four from purebred Landrance and four from purebred Large White) were used as blood donors in this study. Blood monocytes were isolated from these four breeds and the time-course experiment was carried out as outlined in Section 2.3. At each time point, cells were fixed, permeabilized and stained. The fluorescing areas of the capsid proteins with time inside cells of these four breeds were quantified and compared.

### 2.9. Statistical Analysis

Data were analyzed with GraphPad Prism 5 software (GraphPad Software, LaJolla, CA, USA). Treatments were compared by one sample *t*-test after Shapiro-Wilk test. For the kinetics study, differences were assessed by Kruskal-Wallis method; Difference between different breeds were revealed by the area under curve (AUC), followed by the ANOVA with Turkey’s post-test. All results represent means ± SD. Results with a *p* value of <0.05 were considered statistically significant.

## 3. Results

### 3.1. The Uptake and Disintegration of PCV2 Capsids in Blood Monocytes

**Figure 4:**
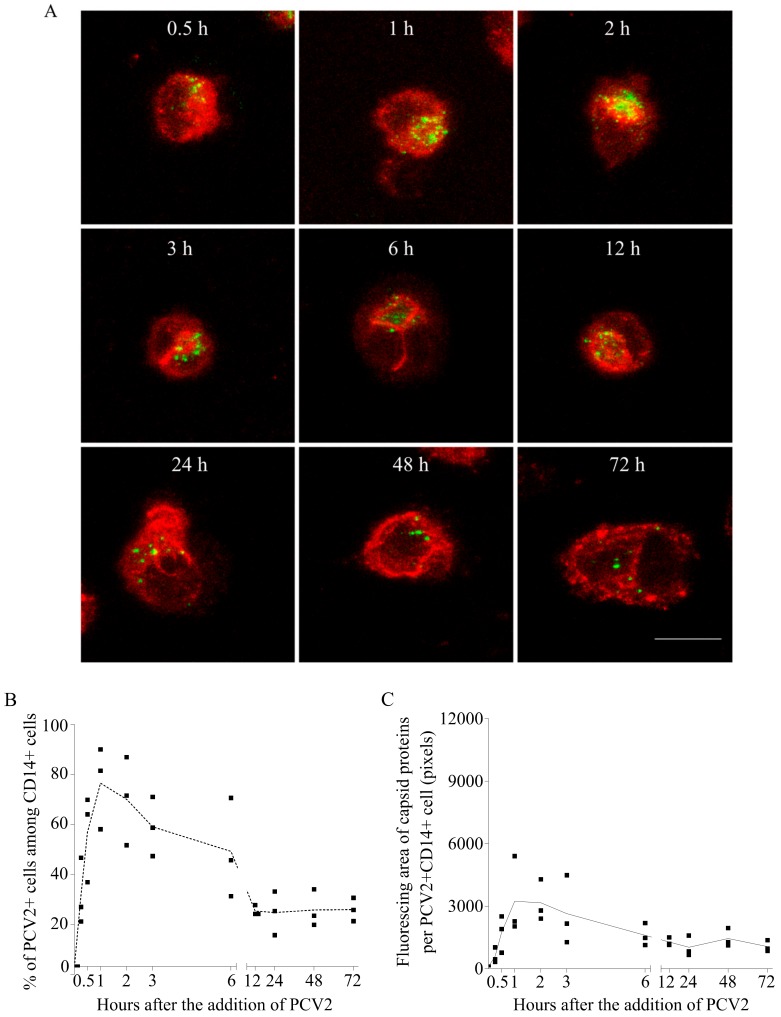
PCV2 particles were taken up by blood monocytes and were then degraded to a certain level. (**A**) Confocal images at selected time points, with cells in red (CD14 staining) and PCV2 capsid proteins in green. Bar = 10 µm. (**B**) Kinetics of the percentage of cells that have taken up PCV2 capsids at each time point. (**C**) Kinetics of the fluorescing area of capsid proteins at each time point. Data were obtained from three pigs (hybrid Piétrain × Hypor Libra).

To understand what PCV2 virions undergo in monocytes, the kinetics of viral capsid proteins inside cells was established, by performing a time-course experiment as outlined in Section 2.3, followed by a double immunofluorescence staining of the capsid protein and the cells. As shown by the confocal images, PCV2 antigen-positive monocytes were detectable at all the time points, and the fluorescent signals of the capsid protein changed with time (Figure 4A). The percentage of monocytes with internalized capsids was calculated (Figure 4B). It rose fast, reaching a maximum of 73% at 60 min. Moreover, within individual PCV2-positive monocytes, the fluorescing area of the internalized capsids increased fast as well (Figure 4C). This means that more and more monocytes were continuously taking up PCV2. After removal of the inoculum, the proportion of PCV2 capsid-positive monocytes dropped to 22% until 12 h, along with a 76% decrease of the fluorescing area per positive cell (relative to the peak value). This shows that monocytes were disintegrating the internalized particles with time. However, the persisting fluorescent signal in certain monocytes afterwards indicated that the capsid proteins could not be completely cleared, and that 70% of the viral antigen-positive monocytes were able to fully disintegrate the capsids, whereas 30% of the positive monocytes had problems with a total clearance.

### 3.2. PCV2 Is Taken up by Blood Monocytes via Clathrin-Mediated Endocytosis

**Figure 5:**
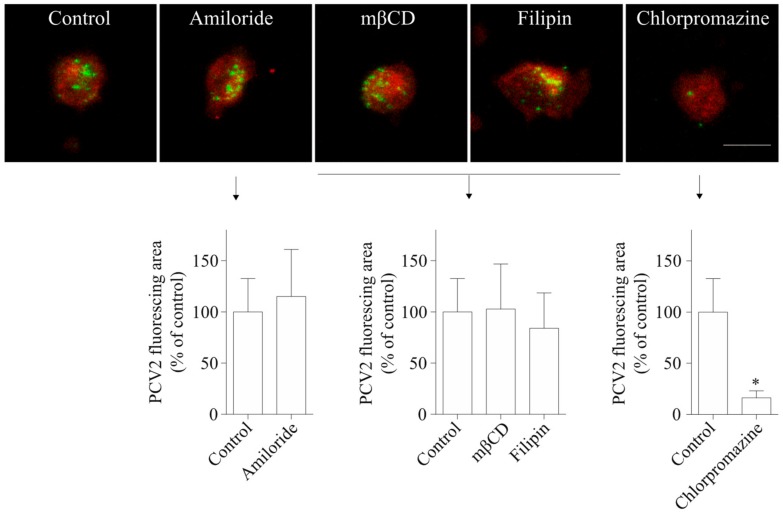
PCV2 entry into blood monocytes occurs via clathrin-mediated endocytosis, and not via macropinocytosis or caveolae-mediated endocytosis. Cells were pre-treated with amiloride (100 µM), two sterol-binding drugs [mβCD (10 mM) and filipin (5 µg/mL^−1^)], or chlorpromazine (1 µM), prior to PCV2 addition for 1 h at 37 °C to allow the virus to internalize. The internalized PCV2 particles were stained in green while monocytes were stained in red for all groups. Bar = 10 µm. The fluorescing area of the internalized PCV2 particles was measured with ImageJ and was calculated as a percentage relative to that obtained in the untreated control. Data were obtained from three pigs (hybrid Piétrain × Hypor Libra) and represent mean ± SD. * *p* < 0.05.

To investigate PCV2 entry routes into blood monocytes, chemical inhibitors were used to treat monocytes before and during PCV2 incubation. The staining and quantification results in Figure 5 showed that cells of all treatment groups took up PCV2, but that the degree of viral uptake was affected by some inhibitors. Amiloride, a specific inhibitor of macropinocytosis, did not lead to any reduction of the PCV2 uptake in monocytes. Also, mβCD that removes cholesterol from the cellular membranes and filipin III that sequesters cholesterol within the membranes, leading to the inhibition of caveolae-mediated endocytosis, did not result in a significant decrease of the PCV2 uptake in monocytes. In contrast, chlorpromazine, a specific inhibitor of clathrin-mediated endocytosis, reduced the uptake of PCV2 by 84 ± 7%. Therefore, it can be concluded that blood monocytes use clathrin-mediated but not caveolae-mediated endocytosis or macropinocytosis to take up PCV2.

### 3.3. The Uptake of PCV2 by Blood Monocytes Requires Actin and Dynamin

**Figure 6:**
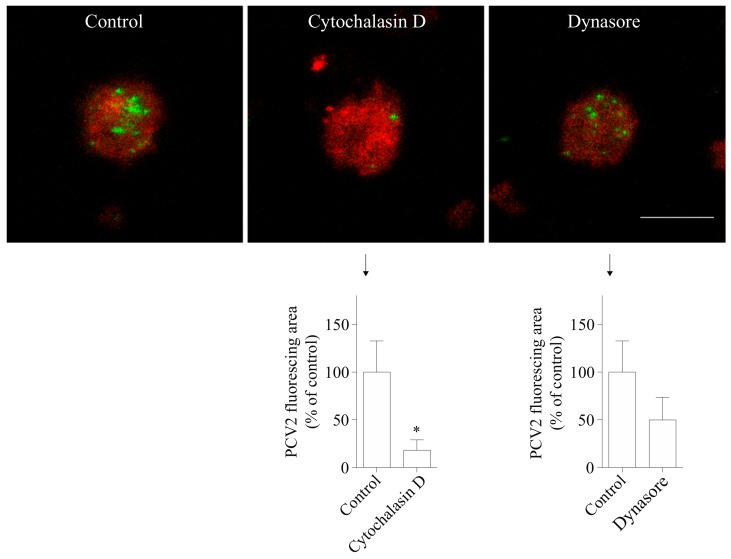
PCV2 entry into blood monocytes is actin-dependent and dynamin is involved. Cells were pre-treated with cytochalasin D (10 µM) or dynasore (100 µM), prior to PCV2 addition for 1 h at 37 °C to allow the virus internalization. The internalized PCV2 particles were stained in green while monocytes were stained in red. Bar = 10 µm. The fluorescing area of the internalized virus particles was measured with ImageJ and was calculated as a percentage relative to that obtained in the control. * *p* < 0.05. Data were obtained from three pigs (hybrid Piétrain × Hypor Libra) and represent mean ± SD.

A growing body of work supports a role for the actin cytoskeleton in clathrin-mediated endocytosis [26,27,28]. In addition, previous studies showed that actin is required in PCV2 infection of 3D4/31 cells and epithelial cells [19]. To test whether actin is involved in the uptake of PCV2 by blood monocytes, cells were pretreated with cytochalasin D, an inhibitor of actin polymerization. The results showed that the green fluorescent signal of the cytochalasin D-treated group was 82 ± 11% lower than that of the control group (Figure 6). Thus, actin is highly involved in the PCV2 uptake in monocytes.

The large GTPase dynamin II is required for pinching off endocytic vesicles from the plasma membrane during endocytosis [29]. We used dynasore which inhibits the activity of dynamin-1 and dynamin-2 to study the requirement of dynamin in PCV2 entry [30]. The results demonstrated that the treatment of cells with dynasore reduced the fluorescent signal of the internalized virions by 50 ± 24% (Figure 6), indicating its inhibitory effect on the uptake of the virus. Therefore, dynamin is involved in the uptake of PCV2 by blood monocytes.

### 3.4. PCV2 Traffics Along the Endosome-Lysosome Pathway

**Figure 7:**
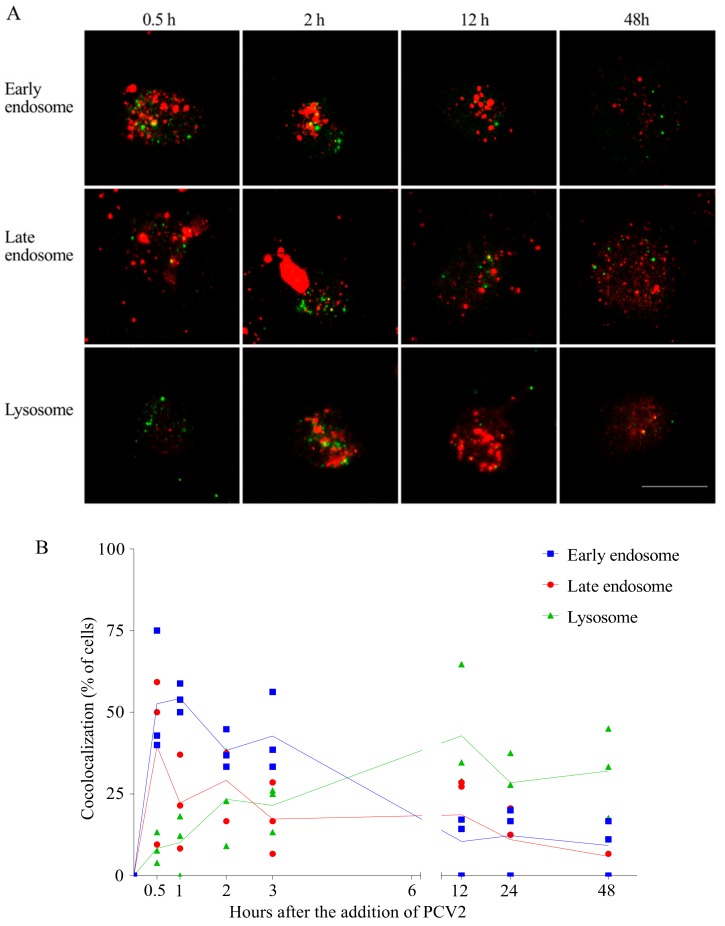
PCV2 capsids traffic along the endosome-lysosome pathway. PCV2 was added to monocytes and samples were collected at different time points after virus addition. Cellular compartments and viral proteins were stained with specific antibodies against early endosomes (EEA1), late endosomes (Rab7), lysosomes (LAMP-1) and capsid protein (94H8). Alexa Fluor 594 or Texas Red-labelled secondary antibodies were used to visualize the cellular compartments (red), while Alexa Fluor 488-labelled secondary antibodies were used to visualize PCV2 capsid proteins (green). (**A**) Confocal immunofluorescence images showing colocalization of PCV2 capsids with the cellular compartments. Bar = 10 µm; (**B**) Kinetics of the percentage of virus positive cells showing colocalization. At least 10 fields were analyzed at each time point. Data were obtained from three individual pigs (hybrid Piétrain × Hypor Libra).

It is known that the clathrin-mediated internalization goes over early and late endosomes [31]. After being internalized via clathrin-mediated endocytosis, PCV2 capsid proteins move to a region close to the nucleus. This is the region where the lysosomes are situated. This led to the hypothesis that PCV2 traffics through the early and late endosomes to the lysosomes in monocytes. In order to examine this hypothesis, a colocalization assay was performed using antibodies against the capsid protein, and against EEA1 (marker for early endosomes), Rab7 (marker for late endosomes) and LAMP-1 (marker for lysosomes). The capsid protein was immunostained green and the cell organelles were immunostained red. Colocalization was defined as any observed merge of the green fluorescent viral protein with the red fluorescent cell organelles (Figure 7A). As shown in Figure 7B, the colocalization of viral protein and early/late endosomes rose fast during the first 30 min. At 30 min post adding the virus, many of the EEA1/Rab7-positive endosomes contained virus particles, which was found in around 50% of the monocytes. The green spots at the periphery of the cell represent viruses that were sticking to the cell but were not yet internalized. These percentages of colocalization gradually dropped in the next 3 h, which is concurrent with the slow rise of the virus-lysosome colocalized cells (25%). This indicated a transfer of the virions from early/late endosomes to lysosomes. This migration to lysosomes went on until 12 h, when approximately 50% of the cells had a virus-lysosome colocalization. However, some of the viral proteins were still colocalized with Rab7-positive endosomes, indicating a slow but continuous exit of the virus from the late endosomes. Afterwards, the percentage of cells with colocalization generally dropped for all these three compartments, which was possibly due to the degradation of PCV2. At 48 h, around 32% of the cells ended up with non-degradable capsid proteins in their lysosomes. This demonstrates a long stay of virus in lysosomes. Not all virus colocalized with cell organelles. Therefore, it is believed that PCV2 may be transferred to a region/organelle that is not recognized by the markers. We concluded from these results that PCV2 capsid proteins traffic along the endosome-lysosome pathway, are partially degraded and end up in lysosomes.

### 3.5. PCV2 Genome Persists in Blood Monocytes in Its Complete Form

**Figure 8:**
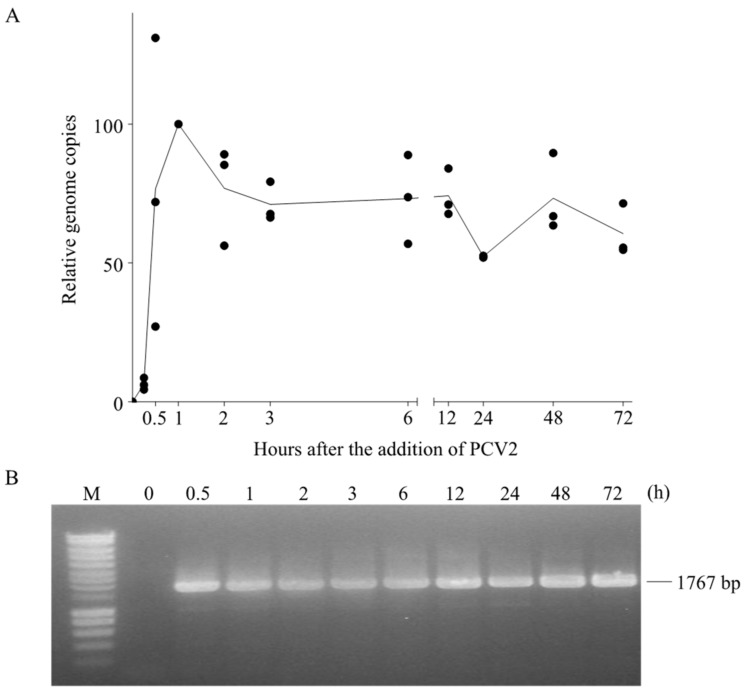
Fate of the PCV2 genome in blood monocytes. PCV2 was added to monocytes and samples were collected at different time points after virus addition. Afterwards, cells were subjected to DNA extraction. (**A**) The number of PCV2 genome copies at each time point was quantified by qPCR and expressed as a relative percentage to that at 1 h; (**B**) The agarose gel electrophoresis image of PCR amplification results of the full-length PCV2 genome at each time point. The PCR was performed with the primer set wgPCV2-fw/rev which amplifies the full length of PCV2 genome. Data were obtained from three individual pigs (hybrid Piétrain × Hypor Libra).

Since PCV2 went through the endosome-lysosome pathway and the capsids were partially disintegrated during this traffic in monocytes, we next investigated what happened with the viral genome. qPCR was performed to quantify the viral genome copies at each time point. As shown in Figure 8A, the number of viral genome copies increased fast and peaked at 1 h, which agrees with the uptake of virions inside the monocytes. After removing the inoculum, it dropped to 70% relative to the original number at 1 h, followed by a steady state until 12 h and with some fluctuations until 72 h. This suggested a persistence of viral genomes in blood monocytes.

Since the qPCR only amplifies a 226 bp of the ORF1 gene region, we further questioned whether the presence of PCV2 genome in monocytes is in its complete form or in digested fragments. Therefore, we used the previously extracted DNA at different time points as templates, and performed a conventional PCR with the primer set wgPCV2-fw/rev (Table 1), aiming for the amplification of the full-length viral genome [25]. Interestingly, we found bands that were equal to the full length of the PCV2 genome in all samples (except the sample at 0 h) (Figure 8B). This means that the complete viral genome persisted in monocytes. This raised two possibilities: (1) the PCV2 genome arrives in the lysosomes together with the capsid proteins, but the porcine lysosomal nucleases fail to digest the single-stranded circular PCV2 genome after disintegration of the PCV2 capsids; (2) the PCV2 genome is released from the capsid in the endosomes and escapes from the endosomes while the empty capsids become transported to the lysosomes. By doing so, the genome is protected from the lysosomal digestion.

### 3.6. Deoxyribonuclease II (DNase II) from Porcine Lysosome can Degrade PCV2 Genome

**Figure 9:**
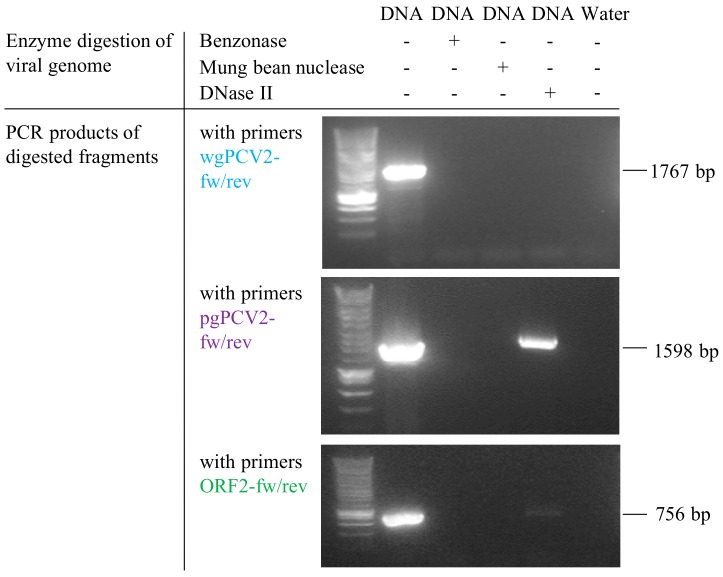
Analysis of PCV2 genome digested with DNase II, mung bean nuclease and benzonase. The agarose gel electrophoresis images showed PCR amplification results of nuclease-digested products. PCR with the primer set wgPCV2-fw/rev, amplifying PCV2 full genome, showed no bands among the three groups treated with nucleases; PCR with the primer set pgPCV2-fw/rev, amplifying a 1598 bp fragment excluding the assumed DNase II cleavage site, clearly showed the corresponding band with the group treated with DNase II and not with those treated with mung bean nuclease or benzonase; PCR with primers ORF2-fw/rev, amplifying PCV2 ORF2 gene including the assumed DNase II cleavage site, showed no band among the three nuclease-treated groups.

DNase II is the primary acidic endonuclease active in the lysosome, which can degrade any DNA that is not effectively shielded by capsid proteins [32]. It exhibits a strong cleavage preference for the site AGAGGA [33], and interestingly, this site is found in the ORF2 gene region (position 461–466, start of ORF2 gene as the origin). To test whether this DNase in lysosomes can function on the circular PCV2 genome or not, a commercial DNase II isolated from porcine spleen was used to incubate with the viral genome extract. In parallel, the viral genome extract was also incubated with mung bean nuclease and benzonase as controls. After incubation, the resulted products were purified for DNA. Then, these DNAs and untreated PCV2 genome were used as templates for further PCR amplification with three sets of primers (Table 1) targeting at different regions of the PCV2 genome, as illustrated in Figure 2. PCR with primers wgPCV2-fw/rev amplified the full PCV2 genome (1767 bp) [25]. As shown in Figure 9, the corresponding band was found in the group of non-treated DNA however not in the enzyme-treated groups. This means that all three nucleases cleaved the PCV2 genome. PCR with primers pgPCV2-fw/rev amplified a 1598 bp fragment that excludes the assumed cleavage site AGAGGA [23]. The expected band was found both in the non-treated group and the DNase II-treated group while not in the other two groups. PCR with primers ORF2-fw/rev amplified the complete ORF2 gene [23], which includes the site AGAGGA. Unsurprisingly, only the non-treated group showed the expected band. From these results, it can be concluded that the DNase II degrades the single-stranded circular PCV2 genome, possibly by a single-strand cleavage (nicking) mechanism at the site AGAGGA. This demonstrates that the PCV2 genome escapes from the endosome-lysosome pathway to avoid being degraded.

### 3.7. Blood Monocytes Differ in the Ability to Take up and Disintegrate PCV2

**Figure 10:**
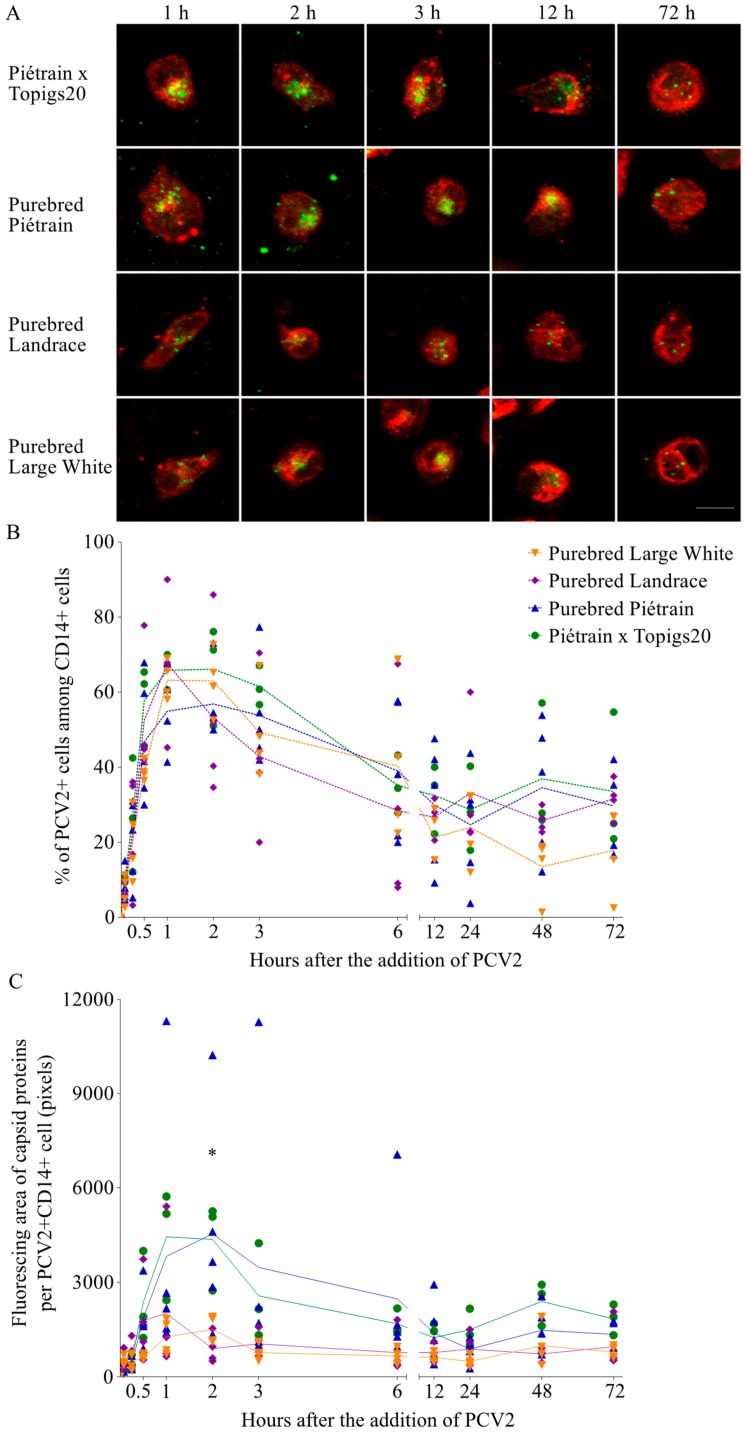

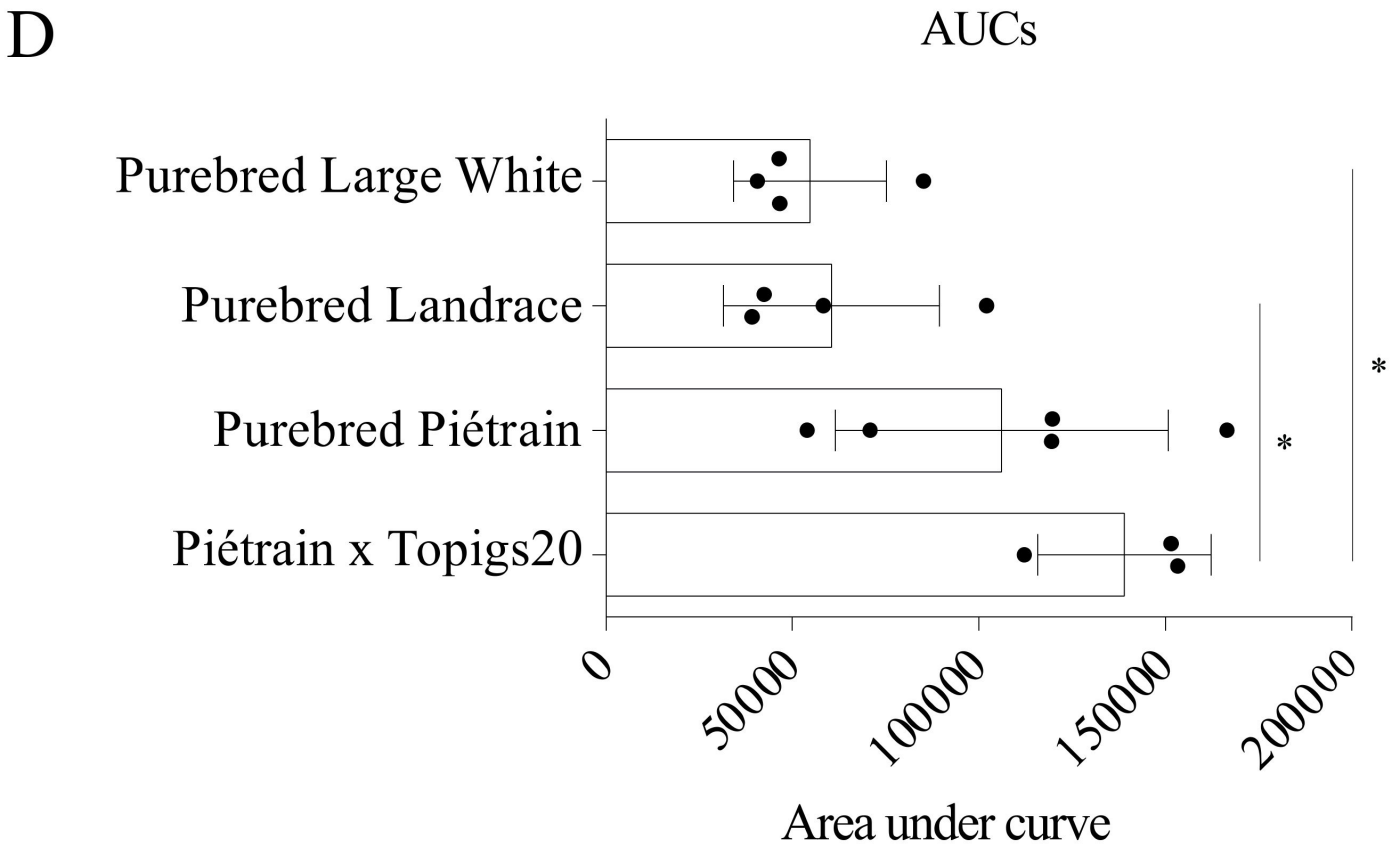
Blood monocytes from different pig breeds take up and degrade PCV2 particles to a certain level, with the purebred Piétrain and the hybrid Piétrain × Topigs 20 showing a higher degree of uptake and disintegration than the purebred Landrace and Large White. (**A**) Representative confocal images at selected time points of the PCV2 capsid protein in monocytes from different breeds. Bar = 10 µm; (**B**) The percentage of PCV2 capsid positive cells at each time point was calculated from 10 randomly selected fields; (**C**) The fluorescing area of the capsid proteins at each time point was calculated with ImageJ. Data were obtained from three pigs of hybrid Piétrain × Topigs20, five pigs of purebred Piétrain, four pigs of purebred Landrace and four pigs of purebred Large White. * *p* < 0.05 (Kruskal-Wallis method); (**D**) The Area Under Curve (AUC) of each animal was calculated. Statistical difference (*p* < 0.05) was demonstrated between Piétrain × Topigs20 and purebreds Landrace and Large White by the one-way ANOVA with Turkey’s post-test.

Monocytes were isolated from four different pig breeds: Hybrid Piétrain × Topigs20 and purebreds Piétrain, Landrace and Large White. The ability of monocytes from these breeds to take up and disintegrate PCV2 virions was evaluated and compared (Figure 10A,C). The kinetics of the percentage of viral positive cells did not differ significantly among breeds (Figure 10B). At 1 h, around 60% of monocytes could take up PCV2 virions. Afterwards, this dropped to approximately 30% and fluctuated around this level. At each time point, the amount of internalized viral proteins in monocytes of the four breeds was quantified (Figure 10A,C). The amount of the incoming capsids peaked at 1 or 2 h in monocytes of all breeds. However, monocytes from hybrid Piétrain × Topigs20 and purebred Piétrain internalized two times more virus capsids than cells from purebreds Landrace and Large White. This demonstrated a stronger ability for PCV2 uptake of cells from the former two breeds than the latter ones. Afterwards, for hybrid Piétrain × Topigs20 and purebred Piétrain, the amount of internalized capsids decreased fast to 25% of the peaked value, indicating 75% of the internalized capsids was disintegrated at 24 h. However, for purebreds Landrace and Large White, only 50% of the internalized capsids was destroyed, indicating a weak ability to disintegrate viral capsids of cells from these two breeds. Furthermore, the area under curve (AUC) was calculated for each individual animal and presented in Figure 10D. Based on these results, we concluded that monocytes from hybrid Piétrain × Topigs20 and purebred Piétrain show a stronger ability to take up and disintegrate viral capsids than cells from purebreds Landrace and Large White.

## 4. Discussion

The monocyte/macrophage lineage cells are generally considered as one of the main target cells for PCV2, based on the fact that PCV2 antigens and nucleic acids could be easily and frequently detected in these cells in vivo [9,10,11,12,13,14,15]. In vitro, the internalization and disassembly of PCV2 in the artificial monocytic cell line 3D4/31 or in the PBMCs were described [11,16,19]. However, the precise mechanism of how PCV2 enters and traffics in primary porcine monocytes and what the viral outcome is were unknown. In the present study, we demonstrated that upon addition of PCV2 to monocytes, virus particles were rapidly internalized, followed by a slow disintegration to a residual level. Then we defined that the fast entry of PCV2 into monocytes occurred via clathrin-mediated endocytosis, and not via caveolae-mediated endocytosis or macropinocytosis. After entry, the internalized capsids followed the endosomal trafficking pathway, ending up in lysosomes, while viral genomes largely escaped from the pathway to avoid degradation. Finally, we demonstrated a breed difference in the ability of monocytes to take up and disintegrate PCV2.

In the kinetic study of the fate of PCV2 capsid proteins in monocytes, samples from all time points were stained and then processed with confocal microscopy at the same time to avoid operational variation. Scanning through PCV2 antigen positive cells at 0.71 µm intervals confirmed the exclusively cytoplasmic localization of PCV2 particles, which has been reported extensively in vivo and in vitro [11,12,13,15]. PCV2 entry into monocytes was found to follow a time-dependent manner within 1 h which is the same as found in the monocytic cell line 3D4/31 cells [19]. At 1 h after the addition of the virus, the internalized virions showed a dispersed pattern. 60% to 70% of monocytes internalized PCV2 particles. This is more efficient and faster compared with the internalization of PCV2 VLPs into 3D4/31 cells, where only 17% of antigen-positive cells were obtained at 1 h and a maximum of 47% at 6 h [19]. Moreover, almost no virus particles were found sticking on the membrane of monocytes at 2 h, indicating a complete internalization of PCV2 particles; while for 3D4/31 cells, only some of the cells were able to fully take up membrane-bound virus particles [19]. This inefficiency of 3D4/31 cells to internalize PCV2 could be due to the different characteristics of the cells. The 3D4/31 cell line was established by immortalizing primary porcine alveolar macrophages with SV40 large T antigen [34]. This immortalization partly altered the characteristics of the primary cells. For instance, the 3D4/31 cell line showed no phagocytic activity in serum-free culture. Only after adding porcine serum to the culture, an inefficient phagocytic activity was demonstrated in a maximum of 50% cells at 18 h. This inefficiency can somehow explain the difference in the speed of virus internalization. Despite the difference, the entry pathway of PCV2 into both cell types occurs via clathrin-mediated endocytosis and requires actin [19]. This pathway is also used by PCV2 to enter the epithelial cell lines PK15, SK and ST and dendritic cells [35,36]. Apart from PCV2, clathrin-mediated endocytosis is used by a large number of other viruses for entry as well, such as porcine reproductive and respiratory syndrome virus, adenovirus, hepatitis C virus, influenza virus [37,38,39,40].

After PCV2 entry into blood monocytes, the dispersed viral fluorescent signal at 1 h became dense and concentrated at 2 h or 3 h, possibly by the fusion of different vesicles carrying PCV2 capsids. Afterwards, the fluorescent signal of viral capsids gradually became weak, concomitant with a decreased percentage of PCV2 antigen-positive cells. The weaker fluorescent signal can be explained by the decreased intact antigen epitopes for the antibody binding. The monoclonal antibody 94H8 produced in our lab was used throughout this study [23]. This antibody was selected because it recognizes a small epitope [41]. The destruction of this small epitope is therefore a strong evidence of the disassembly of PCV2 virions and the follow-up degradation of the viral capsid protein. Interestingly, at 1 h, the internalized capsid protein stained with this antibody gave a brighter fluorescent signal than that stained with the monoclonal antibody 38C1. The mAb 38C1 produced in our lab targets a bigger epitope than that of the mAb 94H8 [23,41]. Thus, the weak signal of the capsid protein stained with the mAb 38C1 may result from the disintegration of this big epitope but not of the small epitope recognized by the mAb 94H8, indicating that the disintegration of the capsid protein possibly already started at 1 h. Further work needs to be done with antibodies targeting different epitopes to have a better understanding of this issue.

Although PCV2 virions were disassembled, this was not a complete disintegration, as indicated by the consistently existing fluorescent signal of viral proteins in the cytoplasm of certain cells from 24 h to 72 h. In other words, the residual viral capsids persisted until the end of this experiment. This persistency of PCV2 in monocytes has been reported before. In monocytes, pulmonary macrophages and monocyte-derived macrophages, it was demonstrated that PCV2 can bypass the normal degradative mechanisms of the monocytes and persist without the detectable reduction in the infectious titer or the number of PCV2 target DNA molecules [11]. In dendritic cells, PCV2 was found to persist without producing virus progeny and viral replicative intermediates [16]. Not surprisingly, both authors concluded that no degradation of PCV2 virions happened since the viral infectivity remained constant during the experiments. Nonetheless, the author also found it incredible that the internalized PCV2 in dendritic cells was not degraded as expected, since most processes of endocytosis affected by dendritic cells lead to the final degradation of the internalized material. However, both authors overlooked the early-degradation events of PCV2 interaction with monocytes, due to the fact that they started sampling at 24 h when the degradation process was already finished and the persistency process started. In the present study, we visually by confocal microscopy showed a quick degradation of PCV2 capsid protein in blood monocytes before the ultimate viral persistence was observed.

This degradation of PCV2 capsids was further confirmed by the colocalization of the viral protein with the early/late endosomes and lysosomes. This is reasonable since it is known that the clathrin-mediated pathway leads to early and late endosomes [31]. During the first 30 min, the virus was found to be colocalized with the early/late endosomes. However, in contrast to the rapid internalization, the complete transit of the incoming virus through early sorting endosomes generally took several hours. Between 3 h and 12 h, when the viral fluorescent signal decreased significantly, virus capsids were found to be increasingly colocalized with the lysosomes. In the meanwhile, the colocalization of virus and the late endosomes was also observed. Although the lysosomes are regarded as the main cellular compartment for degradation [42], we cannot rule out the possibility that, the degradation of virus capsids was already initiated in late endosomes. This hypothesis needs to be further investigated by evaluating the effect of inhibiting lysosome acidification on the fate of incoming capsids. Even at 12 h, a few virus particles were detected to be colocalized with the late endosomes. This is possibly due to a slow exit of virus from the late endosomes to the lysosomes, which was also found in the trafficking of endocytosed products in rat liver endothelial cells [43]. Another possibility is the retrograde transport of non-degradable material from lysosomes to late endosomes [44]. From 24 h onwards, most of the capsid proteins were found to be colocalized with the lysosomes, meaning that the degradation products, as well as non-degradable material, finally ended up in the lysosomes. In this regard, the lysosomes may serve as the storage compartment for slowly and/or non-degradable material. Interestingly, some of the capsid proteins were not colocalized with any of the three organelles, which may be transferred to other organelles or escape to the cytosol. Markers of other organelles like recycling endosomes can be used in the future to study where these viral proteins locate.

Since the viral capsids experienced a partial degradation in monocytes, and finally ended up in the lysosomes, this left the viral genome unshielded and maybe exposed to the dangerous lysosomal environment. With the qPCR that amplifies a 226 bp in the ORF1 gene, we could consistently detect this genomic fragment at all time points through the experiment. This result justified the presence of this fragment in monocytes. We next questioned the survival status of the full-length genome. To answer this question, we performed an additional PCR with primers wgPCV2-fw/rev which were designed to amplify the complete viral genome [25]. Interestingly, the band corresponding to the full length of PCV2 genome was found in all samples. This demonstrated PCV2 genome survived in monocytes as a complete form instead of digested fragments. The persistence of viral genomes in monocytes was also found for African swine fever virus, visna-maedi virus and human adenovirus type 5 [45,46,47], although the exact nature is unknown. This persistence of PCV2 genome may contribute to the evolution of PCV2, since putative recombination events were suggested both in vivo and in vitro [48,49]. Moreover, the two-copy tandemly cloned genomic DNA of PCV2 was demonstrated to be infectious when injected directly into the liver and lymph nodes of pigs [50]. It needs to be further investigated whether the persisting PCV2 genome in monocytes is infectious or not.

Based on the fact that PCV2 capsids are disintegrated in the endosome-lysosome system, and that the complete viral genome stays in monocytes, we generated two hypotheses: (1) the PCV2 genome arrives in the lysosomes together with the capsid proteins, but the porcine lysosomal nucleases fail to digest the single-stranded circular PCV2 genome after disintegration of the PCV2 capsids; (2) the PCV2 genome is released from the capsid in the endosomes and escapes from the endosomes while the empty capsids become transported to the lysosomes; by doing so, the genome is protected from the lysosomal digestion. We proceeded to investigate the first hypothesis. DNase II, the primary nuclease in monocytes, was used to incubate with PCV2 genome in vitro under appropriate conditions [24,32]. The products were evaluated by PCRs with three sets of primers targeting different regions of the viral genome (Figure 3) [23,25]. The results demonstrated that DNase II in lysosomes is able to digest the circular ssDNA of PCV2, possibly by nicking at site AGAGGA in the ORF2 region. This supports the second hypothesis that PCV2 genome escapes from the endosome-lysosome system to avoid being degraded. In many cases, endosomal escape is achieved through penetration of the endosomal membrane either by pH-induced conformational changes of the capsid proteins, or by proteolytic cleavage of viral proteins by acid-dependent endosomal proteases [51]. Therefore, we suggest that the acidic milieu of the endosomal compartment triggers conformational changes in PCV2 capsids, promoting PCV2 genome release and subsequent endosomal escape. This phenomenon is also found in the infection process of other viruses [52,53,54]. In the case of picornaviruses, the viral RNA escapes from the endosomes and enters into the cytosol, leaving the capsid behind in the endosome [54]. This might be the same for PCV2, which suggests that lysosomal localization of fluorescent viral signal may be empty viral capsids.

Apart from the endosomal-escape theory, a small decrease in the number of PCV2 genome copies was observed upon virus internalization. This may be due to a fast digestion, in late endosomes or some pre-existing lysosomes, of the viral genomes which are not well-shielded/protected by the capsids. Naked viral genomes exist in the virus stock we used. Since the virus stock is produced in PK-15 cells, there are always some handicapped viruses which are not intact and not fully-packaged, and/or some free virus genomes which are not packaged at all. The easy digestion of the viral genome from these handicapped virus particles may explain the small drop in the amount of the viral genome shortly after the uptake. Interestingly, from 24 h onwards, a slight increase of virus genome copies was found. This increase can be explained by the probable misuse by PCV2 of the DNA polymerase produced by monocytes in response to damage to their DNA [55]. It is well known that with the help of cellular DNA polymerase, PCV2 can complete its replication cycle. Indeed, some originally adherent monocytes were observed to detach from the culture plates from 48 h onwards, indicating a cell-suffering environment of the in vitro culture. This may have initiated the self-repair machine. In addition, this result is in line with the findings from other studies. In dendritic cells, a similar increase in the number of PCV2 genome was observed with time after PCV2 inoculation under certain conditions, suggesting a tendency of dendritic cells to support a limited PCV2 replication [16,56]. Moreover, the replicative form of PCV2 genome was demonstrated in lysozyme-positive cells, further indicating that at least a certain fraction of macrophages could support PCV2 replication [57]. A more direct proof is the detection of nuclear localization of PCV2 rep protein in macrophages both in vivo and in vitro [9,58], since this protein is not present in the mature virion and therefore has to be synthesized during virus replication. In contrast, the incapability of PCV2 replication in monocyte-macrophage lineage cells without proper stimulation were also reported [59,60]. Further research needs to be done to clarify the current picture on this issue. The ultimate persistency of the viral genome in monocytes may explain the in vivo phenomenon that PCV2 nucleic acids are consistently detected in pigs suffering from PMWS or experimentally inoculated with PCV2 [10,12,13,14]. It could be interesting in the future to find out how the viral genome escapes from the endosome-lysosome system of monocytes and what the consequences are for the pigs.

In the last part of this study, we evaluated the effect of the genetic factor in the ability of monocytes to take up and disintegrate PCV2. We compared the kinetics of the amount of viral capsids in monocytes from four different pig breeds. It turned out that the main difference does not lie in the percentage of the cells that take up PCV2 (60–70%), but instead in the amount of PCV2 capsids that are engulfed per active cell in the PCV2 uptake. Indeed, monocytes, known as scavenger cells, are responsible for cleaning up foreign material out of the body. The more virus particles they take up and degrade, the cleaner the body environment. Moreover, if most virus is internalized by monocytes, there will be less free virus left for infection of lymphoblasts, the fully susceptible targets of PCV2 [9,17,18]. Specifically, we defined that monocytes from purebred Piétrain and hybrid Piétrain × Topigs20 were able to take up twice more PCV2 particles than those from purebreds Landrace and Large White. Moreover, the disintegration of the internalized virus particles was more efficient with the former ones (75% of the total) than the later ones (50% of the total). This in vitro finding corresponds to the field observations that Piétrain pigs are less likely to develop PCV2-associated diseases. Moreover, a controlled study from Opriessnig et al. (2009) determined that Landrace pigs were more susceptible to PCV2-associated lesions than Piétrain pigs [22]. Therefore, our findings may in part explain the difference in genetic susceptibility of PCV2. To further clarify the whole picture, more detailed genetic work needs to be done.

Nowadays, PCV2-associated diseases are well controlled by vaccines. Indeed, vaccination against PCV2 seems to be successful in reducing losses on affected farms. However, vaccinated pigs still seem to harbor and potentially shed PCV2. This sneaky virus has imperceptibly emerged in our daily life. The dietary consumption of PCV-harboring pork leads to the detection of PCV DNA in human stool, which shares 99% overall genome nucleotide similarity with PCV2. Around 5% of the stool samples from adults in the United States were detected to be PCV positive, and even stunningly, 70% of the store-bought pork products also frequently contain PCV sequences. Although no transmission experiment was performed from pig to human, the transmission of PCV2 from one pig to another through consumption of meat was already shown [61]. In addition to PCV DNA in consumable products, some commercial rotavirus vaccines were also detected to contain PCV DNA [62], and not surprisingly, PCV DNA was found in feces from children who received live rotavirus vaccines [63]. It is not known whether the PCV found in human samples can replicate in their human host in vivo. So far, no antibodies to PCV2 were detected in healthy human blood [64]. However, in human cell lines infected with PCV2 in vitro, viral protein expression and DNA persistence were demonstrated, but luckily the virus could not be passed to new cultures [65]. In conclusion, to the best of our knowledge, no sustainable human PCV infections could be demonstrated. It could be interesting to study if PCV2 DNA may persist in cells of the monocytic lineage and to find out what the consequence is.

In summary, we showed that PCV2 enters blood monocytes fast, followed by a slow disintegration of viral capsids to a residual level. The uptake of PCV2 occurs via a clathrin-mediated endocytosis, which is dynamin- and actin-dependent. After entry, PCV2 traffics through the endosome-lysosome pathway, with the viral genome escaping from the endosomes, leaving the viral capsids partially degraded in the lysosomes. In addition, we proposed that the difference in the ability to take up and disintegrate PCV2 by monocytes may partly contribute to the genetic susceptibility of PCV2 among different breeds. Further unravelling of how the viral genome escapes from the endosome-lysosome system of the monocytes and what role persisting PCV2 genomes play in PCV2-associated diseases may provide new insights into the complex pathogenesis of these diseases.

## Figures and Tables

**Table 1:** Primers used to obtain different regions of PCV2 genome.

**Primer Set**	**Forward Primer (5′ to 3′)**	**Reverse Primer (5′ to 3′)**	**Product**
wgPCV2-fw/rev	ggaagcttcagtaatttatttcatatggaa	ggaagcttttttatcacttcgtaatggtt	1767 bp (whole PCV2 genome)
ORF2-fw/rev	gcgcacttcttttcgttttcag	gaatgcggccgcttatcacttcgtaatggtttttattattca	756 bp (DNase II cleavage site included)
pgPCV2-fw/rev	ggctgtggcctttgktac	tgtrgaccacgtaggcctcg	1598 bp (DNase II cleavage site not included)
